# Supplementary material for: Aeromonas hydrophila infection induces Toll-like receptor 2 (tlr2) and associated downstream signaling in Indian catfish, Clarias magur (Hamilton, 1822)
Source: PeerJ. 2021 Nov 30;9:e12411. doi: 10.7717/peerj.12411 (PMC8641487; doi:10.7717/peerj.12411)
Supplement: Supplemental Information 2 [file peerj-09-12411-s002.docx]

# >Toll like receptor 2 (Tlr2) Indian catfish, Clarias magur

ATAGTTAAGCGAGAAAAGATTACTACAAGCAAGACTTTTATCTACTGGTGTACTTGATAATAACTACCGGTTTTGTTTTGTTTTGTTTTGTTTTTAGTTTTGATTCTACTGTGATGCAAATGCGACACTTCAGGCTGATGTTTTCAGCTTCTCTCCAGAAACAACACACATCCAGCAGGATGAAGGTGCCACTGGCCTTATGTATCTGTTTTAGCCTGACTCTGACTCTGACTCAGACCTCCGAGAGACCAACCTGTAATGACTGCGACGAAGATCATTTCTGCAACTGTCGTGCTAAGGACCTCCACGGTGTCCCCATAGTTCCAGATGATGTTCTTTACCTAGACGTGTCCTTCAACGAGATCGAGTCCATCACTCAGAGGGATCTGACCTGCTACACAGAGCTGAGAAATTTAAAGCTGCAGAAGAACAAACTCAGCACGATCCACAAAGAAGCATTTCATTCCCAAAGTAAACTGGAAGCGCTTGATCTGTCATTCAATAACCTGAAAAACATTTCCTCCCAATGGTTTTCTAATCTTCGGTCCCTGAAACATTTGAACATCTTGGGAAACCAGTACACCACTTTGGGATCCATCGCCTTGTTTCAATTTGTCGAAAACCCCGCGCTGAGAACGTTACAGTTCGGCAACCTTTGGATCAGGGATGTGAAACAGAATTTGCTGCGTAATATTAGACAGCTGGATGAGCTGTCGTTTGTCGGTGGTGTCCTCAGATCATATGAGAATGGAAGCTTCCAGACGATTCAACCCATCAGAGCCGTGTCAGTCAGCCTTTCGCGGTTGTTTCAGGATGATCCAGCACTGGTATCAAAGATCCTTCGAGATGTTTCTCACCCTGAGACATCGCTGACCATTAGAGATGTCTCCCTGGAGACACAAGAACTGATAGAACCCTTAAGAGAGGTGACAGAAGGTGGCACCAGAAGTCTTACCTTTCAAAACATAATCACAACTGACGAGGCAGTCAGCCGCCTTCTGGAGGTTTTGGACGGCTCTCCGGTGTCCTACATCGGCCTTGAGGACATTTGTTTAATAGGTCAGGGCTGGTGGGAAAAGGCGAAGAGGACACACCTAGAAAACCTGCACACGATACATGTCCGCAACATAGAAATCCAGGGCTTCTTCAAATTTAGCAGCATGATACAGTTAGCGTTCCTGTTGAAGCACCTCACCAAGATATCCGTCATCAACTGCACCGTTTTCGTTATTCCCTGCCTGACCAGCTGTTTTCTTAAAAAGGTGGAGTACTTGGACTTGAGCCAAAACCTCCTCTCGGATATCACCATGCAAGAATCCCTGTGCAACGGGGACAGCAAGATGCGCAATATTAACACGCTCAATGTAAGTCACAACTCGCTGAAATCTCTGCAGCTCATGTCCCACCTGGTCACGAGTCTCGACAGGCTGACATCGCTAGACATGAGCCACAACAACTTTGTAAAGATGCCACAGAGTTGCAGCTGGCCGGCAAGTCTCAGGTTTATGAACCTGTCCACTACAAAACTTCACCGCGTAACCCCGTGCCTACCTCTCAGCCTGACCGTGCTGGATTTGAGCCAGAACTTCCTGACAGAGTTCCACCTCCATCTTCCCAACCTTGCGGAGCTCTGGCTTACAGGGAACAGGATTATTGCCCTGCCGGAAGGTGGCCACTTCCCCAGCCTACGCATGCTGTTTATTCAAAGCAACACATTGAACATGTTCAACAAAAGCGACCTGATGGCGTTCCAGTCTCTCCAGGTCTTGGAAGCCGGACATAACAATTTTTTTTGCAGCTGCGATTTCGTAGAATTCTTTCAAGGTTCTATTGACCACTTGATCACTCTGGGGGACGGACATCGCAGCTACATGTGTGACTCTCCGTTCACGTTAAGGGGTCTTAATATAGATACCGCTCAACCGCCAGTCTTCGAGTGCTACATGATCCTGTTAGTATCAGTCATCTGCTCGGTCACCGTCATCGGCGTGATCGCCATCGGGGTCACCTGCCACAAATTCCACATCTTGTGGTACCTGCAGATGATGATCGCGTGGTTAAAAGCAAAGAGTAAACCATCCGTGCAAATGGCGGCGCTACTTTTCGATACGATGCTTTTCGTGTCGTACAGCCAGCACGATGCGCAGTGGGTGGAGGAAATCCTCGTGCCAGAGTTAAAAAGCTCTGAGTCTCCGCTCGCTCTGTGTCTGCACCAGCGGGACTTCCTCCCAGGCCGCTGGATCGCCGACAACATCATCGAGTCCATCGAAAGCAGCTATCGGACCCTCTTTGTCCTGTCGGAGAACTTCGTGACGAGCGAGTGGTGCCGATACGAGCTGAACTTTTCGCATTTTCGGATCATCGACGAGCGCAACGATTCGGCCGTCCCTGATCCTGCTAGAGCCCATCGCCAAGGAGACGATTCCCAAGCGCTTCTGCAAACTGCGCAAAATAATGAACTCCAGGACGTACCTCGAGTGGCCTGAGGACGAAGAAAAGCGAGAGGAATTTTGGCACAATCTCCGAGCTGCACTTAGAAGGGAGGACTCGTGATGCCGATTCTGATACGTCACGGATCACACCTTCATCAGATTTGAAAATAATTACCCGGTTTCATTTTGTCAGTAATTTGAAGTTCTGACAGCGGAGAACCCTTACTTGTTACTTGTGGTGTGTCTACAGCATACGCACTTATTTACCGTTAGAGCTCGTTATTCCTCACCTGATGTACAGATTAGACTCAAACCCAGACTCAACCTCTGATTAAGTTCTGCTCTCAATAAACACAAGCTCTATCTATTTAAGGATCTTCTGCATCGGGGGCTTTCCTTTTTATGGTTAGCTTCCTCATAAAATGGAGAACACACACACACACACATGCACACATCTTATGAGTAATTCTTCAGTTATTCACTCAATGAAACCTTCCTCATGATGGTTAGATTCGTGCTGTCTTGTTTTAATATGATTTTATATTACAGAAGTGGAAAGATTATTAATAATTAACTTGTGAAAATAAACGCAGCGAGGAATCATTTCAAAAAAAAAAAAAAAAAAAAAAAAAAA
